# Fabrication and Characterization of Orodispersible Composite Film from Hydroxypropylmethyl Cellulose-Crosslinked Carboxymethyl Rice Starch

**DOI:** 10.3390/membranes12060594

**Published:** 2022-06-04

**Authors:** Ornanong S. Kittipongpatana, Karnkamol Trisopon, Phanphen Wattanaarsakit, Nisit Kittipongpatana

**Affiliations:** 1 Department of Pharmaceutical Sciences, Faculty of Pharmacy, Chiang Mai University, Chiang Mai 50200, Thailand; karnkamol.t@cmu.ac.th; 2 Department of Pharmaceutics and Industrial Pharmacy, Faculty of Pharmaceutical Science, Chulalongkorn University, Bangkok 10330, Thailand; phanphen.a@chula.ac.th

**Keywords:** orodispersible film, polymer composite, hydroxypropylmethylcellulose, crosslinked carboxymethyl starch, disintegration time

## Abstract

Crosslinked carboxymethyl rice starch (CLCMRS), prepared via dual modifications of native rice starch (NRS) with chloroacetic acid and sodium trimetaphosphate, was employed to facilitate the disintegration of hydroxypropylmethylcellulose (HPMC) orodispersible films (ODFs), with or without the addition of glycerol. Fabricated by using the solvent casting method, the composite films, with the HPMC--LCMRS ratios of 9:1, 7:1, 5:1 and 4:1, were then subjected to physicochemical and mechanical evaluations, including weight, thickness, moisture content and moisture absorption, swelling index, transparency, folding endurance, scanning electron microscopy, Fourier transform infrared spectroscopy, tensile strength, elongation at break, and Young’s modulus, as well as the determination of disintegration time by using the Petri dish method (PDM) and slide frame and bead method (SFM). The results showed that HPMC-CLCMRS composite films exhibited good film integrity, uniformity, and transparency with up to 20% CLCMRS incorporation (4:1 ratio). Non-plasticized composite films showed no significant changes in the average weight, thickness, density, folding endurance (96–122), tensile strength (2.01–2.13 MPa) and Young’s modulus (10.28–11.59 MPa) compared to HPMC film (135, 2.24 MPa, 10.67 MPa, respectively). On the other hand, the moisture content and moisture absorption were slightly higher, whereas the elongation at break (EAB; 4.31–5.09%) and the transparency (4.73–6.18) were slightly lowered from that of the HPMC film (6.03% and 7.03%, respectively). With the addition of glycerol as a plasticizer, the average weight and film thickness increased, and the density decreased. The folding endurance was improved (to >300), while the transparency remained in the acceptable range. Although the tensile strength of most composite films decreased (0.66–1.75 MPa), they all exhibited improved flexibility (EAB 7.27–11.07%) while retaining structural integrity. The disintegration times of most composite films (PDM 109–331, SFM 70–214 s) were lower than those of HPMC film (PDM 345, SFM 229 s). In conclusion, the incorporation of CLCMRS significantly improved the disintegration time of the composite films whereas it did not affect or only slightly affected the physicochemical and mechanical characteristics of the films. The 5:1 and 4:1 HPMC:CLCMRS composite films, in particular, showed promising potential application as a film base for the manufacturing of orodispersible film dosage forms.

## 1. Introduction

The development of orodispersible (also known as orally dissolving, orally disintegrating) films (ODFs) has gained significant attention over the past decade, largely due to their unique characteristics and ease of administration which enabled patient compliance, thus benefiting individuals who experience or suffer from chewing and/or swallowing problems, including pediatrics and geriatrics [1]. ODFs conform essentially to a single unit drug dosage form which, once placed into the mouth, would blend with the saliva and disperse or dissolve readily without the need of water or chewing. The released active drug can then be locally absorbed through the oral mucosal or buccal epitheliums or effortlessly swallowed for systemic action [2]. Several production technologies have been researched and established, including solvent casting, hot melt extrusion, solid dispersion extrusion, rolling, and 3-D printing methods [3,4] such that at present ODFs are projected to become one of the most effective dosage forms for drug delivery [4]. In addition, ODFs can potentially be used for extemporaneous compounding in case the required dosage drug is not commercially available or in the preparation of personalized medicine [5]. In any case, the ODF formulation must fundamentally contain a water-soluble, film-forming polymer or polymer blends with rapid dissolution to release the drug, or a disintegrating/dispersing agent must be included [2].

Cellulose is the most abundant biopolymer in nature and is utilized, in its native/nanofiber form, derivatives, or as a composite with other materials, in a broad range of applications, including textiles, foods, engineering, water treatment, and pharmaceuticals [6,7,8]. Hydroxypropylmethylcellulose (HPMC) is one of the most commonly utilized polymers in the preparation of pharmaceutical thin film as it forms a film with good appearance, tensile strength, and flexibility [9]. Also utilized as a binder and hydrophilic matrix for tablet formulation, however, HPMC is known to navigate the release of a drug in a controlled mode [10], therefore a rapid dissolution is not warranted for an HPMC-based film. A disintegrating agent is usually needed to ensure or facilitate the proper breakdown of the film and the release of the drug. Starch, also an abundant natural biopolymer, is known for and has been applied as a disintegrating agent in the tablet formulation but the amount required for such action is typically in the 10–20% range. In addition, native starch, despite being biodegradable, does not normally produce an acceptable film, but rather requires chemical or physical modification in order to achieve so. Oxidation, hydroxypropylation, and crosslinking are among the chemical reactions employed to improve the film-forming properties of starch [11,12], whereas heat-moisture treatment, pregelatinization, and alcoholic-alkaline and ball milling treatments are reportedly effective physical modifications [13,14,15]. Cross-linked carboxymethyl rice starch (CLCMRS), prepared via the dual reactions of carboxymethylation and crosslinking of native rice starch with sodium trimetaphosphate, exhibited potential as a superdisintegrant for tablet formulation [16]. CLCMRS was also reported to possess good film-forming ability due to the substitution of hydroxyl groups with the larger carboxymethyl moieties capable of reducing the molecular hydrogen bonds along the starch chains [17]. Such substitution increased the flexibility of the network and promoted the formation of film similar to the effect of a plasticizer [18]. 

Cellulose-starch composites have been studied and reported by several research groups but mostly as a way to achieve structural reinforcement [19,20,21]. The combination of HPMC-CLCMRS would theoretically alter the film appearance and texture while enhancing the disintegrating property. It was imperative to investigate how the combination would affect the mechanical properties, e.g., tensile strength, elongation at break, and other pertinent properties of the film. This study incorporated CLCMRS as a disintegrant in the composite films at various ratios and evaluated their physicochemical and mechanical characteristics. 

## 2. Materials and Methods

### 2.1. Materials

Native rice starch (RS) (Lot no. 709161) was purchased from Thai Flour Industry Co., Ltd. (Bangkok, Thailand). Monochloroacetic acid (MCA, CAS No. 79-11-8, Product Code 8004121000) was purchased from Merck (Hohenbrunn, Germany). Sodium trimetaphosphate (STMP, CAS No. 7785-84-4, Product Code 1001229448) was supplied by Aldrich (IL, USA). Hydroxypropyl methylcellulose (Methocel E5LV Premium) was supplied by Rama Production Co.Ltd. (Bangkok, Thailand). 

### 2.2. Preparation and Properties of Cross-Linked Carboxymethyl Rice Starch (CLCMRS)

The preparation of carboxymethyl rice starch crosslinked with sodium trimetaphosphate (CLCMRS) was achieved using the steps and reaction conditions described in the previous study [16]. In brief, monochloroacetic acid (40 g) was dissolved in methanol (MeOH) (254 g) for 10 min. Rice starch (138 g) was then dispersed in the solution, followed by the additions of a solution containing 40 g sodium hydroxide and 40 g water, and sodium trimetaphosphate. The slurry mixture was maintained under reflux conditions at 70 °C for 60 min, after which the pH was adjusted to 7.0 with glacial acetic acid. The modified starch was collected by vacuum filtration (Whatman no.1 filter paper) and washed several times with 80% MeOH until the precipitate test of the filtrate with 1%*w/v* silver nitrate solution was negative. Finally, the product was washed with the AR-grade MeOH and was dried at 60 °C for 24 h in a hot air oven. The dry modified starch was passed through a No.60 sieve and was subsequently subjected to the determinations of degree of carboxymethyl substitution (DS), degree of crosslinking (DCx), amylose content, water solubility/swelling power according to methods previously described [16]. Analyses of moisture, ash, protein, and fat contents in CLCMRS were conducted according AOAC 925.19, AOAC 942.05, AOAC 992.23, and AOAC 920.39, respectively.

### 2.3. Preparation of HPMC-CLCMRS Composite Film

HPMC LV5 (3% *w/v*) was dissolved in heated water (70 °C, 15 min) while stirring at 150 rpm using a magnetic stirrer (Heidolph Instruments, Schwabach, Germany) and allowed to cool to room temperature. Crosslinked, carboxymethyl rice starch (CLCMRS) was prepared as a 3%*w/v* solution by dispersing in unheated water for 30 min. The two solutions were mixed at different ratios according to Table 1, with or without the addition of glycerol, a plasticizer (0, 1.5, 2.5%*v/v*). Each mixture was degassed in an ultrasonic bath (10 min), and then 30 mL was poured on a circular Teflon plate of 12.5 cm diameter, and dried in a hot-air oven at 50 °C. After 16 h, the casted film was removed from the plate and visually inspected. The film was then kept in a humidity chamber (25 °C, RH = 67%) until further analyses were carried out. Pristine HPMC, CMRS, and native rice starch films were also prepared for comparison purposes.

### 2.4. Physicochemical Properties of HPMC-CLCMRS Composite Films

#### 2.4.1. Average Weight

The weight of a 2 × 2 cm^2^ film strip sample was recorded using a Sartorius LA230S analytical balance (Sartorius, Göttingen, Germany). Each sample was tested in triplicate and the average ± SD was calculated. 

#### 2.4.2. Film Thickness and Density

The thickness of each film sample was measured at five different positions, the center and the four corners, by using a digital Vernier caliper (Mitutoyo, Kawasaki, Japan). The accuracy of the instrument was 2.5 mm ± 0.5%. The test was repeated three times, and the average ± SD was calculated. 

The density of each film strip was calculated as the weight divided by the volume (area × thickness). The results were expressed as g/cm^3^ [22].

#### 2.4.3. Swelling Index

The swelling index of ODFs was evaluated according to the method described previously [2], with slight modification. In brief, the film sample of 2 × 2 cm^2^ size was placed on a metal sieve and immersed in 40 mL of artificial saliva solution (8 g NaCl, 0.19 g KH_2_PO_4_, and 2.38 g Na_2_HPO_4_ in 1L distilled water, pH 6.8) under a controlled temperature of 37 ± 1 °C. The sample was weighed every 30 s until the maximum absorption of water was achieved. The weight of the swollen film was recorded. The swelling index was calculated as the ratio between the weights of the film after and before the immersion, respectively.

#### 2.4.4. Moisture Content

The moisture content of the film sample was determined by using an Ohaus MB25 moisture content balance (Ohaus Corp., Parsippany, NJ, USA) equipped with a halogen radiator. Approximately 2 g of the sample was spread on the pan and the accurate weight was recorded. The sample was heated at 105 °C until a constant weight was obtained. The moisture content was then calculated as the difference between the two weights divided by the original weight of the sample [22]. 

#### 2.4.5. Moisture Absorption

A pre-weighed 2 × 2 cm^2^ film strip sample was placed in a desiccator housing a saturated sodium chloride solution, which provided a 75% relative humidity at 30 °C. After 72 h, the film was reweighed and the moisture absorption percentage of the ODF was determined as the difference between the two weights divided by the original weight of the sample. 

#### 2.4.6. Transparency

The transparency of the films was assessed by determining the absorbance of the film by using a spectrophotometer (UV2450, Shimadzu, Kyoto, Japan) at 600 nm [21]. The relative transparency (T value) of the films was calculated by using the following equation;

T value (mm^−1^) = (1/10^A_600_^)/X (1)

where A_600_ was the absorption at 600 nm and X was the film thickness (mm). The greater T value represented the better film transparency. 

### 2.5. Scanning Electron Microscopic (SEM) with Energy-Dispersive X-ray Analysis

SEM studies to visualize the granule surface, shape, and size of the CMRS powder sample and the surface and cross-sectional images of the film samples were conducted by using a JEOL instrument model JSM-5410LV (JEOL, Tokyo, Japan) equipped with a tungsten filament K-type. The acceleration voltage was 15 kV under the low vacuum mode (0.7–0.8 torr). The sample was mounted on a copper stub covered with carbon tape and sputter-coated with gold. The magnification was set at 2000× for modified starch granules and 250× for films. The analysis of the chemical composition of the modified starch was performed by using an energy-dispersive X-ray (EDX) spectrometer (ISIS 300, Oxford Instrument, High Wycombe, UK) [17].

### 2.6. Fourier Transform Infrared Spectroscopy (FT-IR)

The Fourier transform infrared (FT-IR) spectrum of HPMC, CLCMRS, and HPMC-CLCMRS composite films was recorded at room temperature by using a Thermo Nicolet Nexus 470 FT-IR ESP Spectrometer (Thermo Electron Corporation, Waltham, MA, USA) operated at a resolution of 4 cm^−1^. The spectrum was recorded by using a Smart multi-bounce HATR mode in the wavenumber range 4000 cm^−1^ to 400 cm^−1^, with 64 scans [21]. 

### 2.7. Mechanical Properties

The mechanical properties of the films were investigated according to ASTM D882-97 testing standard by using a TA.XT plus texture analyzer (Stable Micro Systems, Surrey, UK) with a 0.5 kN load cell [23]. Intact films were cut into rectangular strips of size 2 × 6 cm^2^ and stored at 25 °C and 58% RH for 48 h before testing. The initial grip separation and the crosshead speed were set to 20 mm and 0.5 mm/s, respectively. Tensile strength (TS, MPa), elongation at break percentage (EAB, %), and Young’s modulus (YM, MPa) of the intact films were determined using the following equations;

Tensile strength (σ) = F_m_/A (2)

Where F_m_ was the maximum stress (N) at which the film broke; and A was the original cross-sectional area of the film specimen (m^2^). The values were expressed in MPa unit.

(3) $$\mathrm{Percent}\;\mathrm{elongation}\;\mathrm{at}\;\mathrm{break}\;(\varepsilon )=\frac{\left(d_{2}-d_{1}\right)}{d_{1}}\;\times \;100$$

d_1_ was the original length of the film between the grips and d_2_ was the length at the point of film rupture

(4) $$\mathrm{Young}’s\;\mathrm{modulus}=\frac{\mathrm{Slope}\;\mathrm{of}\;\mathrm{the}\;\mathrm{stress}-\mathrm{strain}\;\mathrm{curve}\;\times \;\mathrm{initial}\;\mathrm{sample}\;\mathrm{length}}{\mathrm{film}\;\mathrm{cross}\;\sec\mathrm{tion}}$$

The results were expressed in MPa unit [5]. 

### 2.8. Folding Endurance Test

Folding endurance was performed according to ASTM D2176-97a [24] by using a folding endurance tester GT-6014-A (Gotech Testing Machines, Taichung City, Taiwan) with a 200 g load cell. Film strips were equilibrated at 65% RH, 27 °C for 24 h before folding (folding angle 135°, 175 times/min) until they broke. The number of folding times before the crack or break was recorded as the film endurance.

### 2.9. In Vitro Disintegration Test

#### 2.9.1. Petri Dish Method

The disintegration time of the films was determined according to the method reported by Kim et al. [25] with slight modification. The ODF specimens (2 × 2 cm^2^) were placed in a petri dish containing 5 mL of artificial saliva, and the dish was shaken at 80 rpm at 37 ± 1 °C. The time was recorded when the film disintegrated completely.

#### 2.9.2. Slide Frame and Bead Method

This method was slightly modified from the slide frame and the slide frame and ball methods described by Speer et al. [26]. A rectangular cut film of 4 × 4.5 cm^2^ size was locked into a 5 × 5 cm^2^ slide frame exposing a 2.5 × 3.5 cm^2^ window. The assembled frame was placed on a petri dish (5 cm diameter) and a round plastic bead (d = 2 mm, mass = 0.08 g) was placed on the film surface. Distilled water (200 μL, 37 ± 0.5 °C) was dispensed onto the film surface. The time required for the plastic bead to drop into the petri dish was recorded as a disintegration time. 

### 2.10. Statistical Analysis

All tests were performed at least in triplicate and the data are expressed as mean values. Statistical analysis was conducted by using a one-way analysis of variance (ANOVA) in SPSS (version 19.0). Significance tests of means were analyzed using Tukey’s honestly significant difference (HSD) multiple range test at a 95% confidence level (*p* < 0.05). 

## 3. Results and Discussion

### 3.1. Preparation and Properties of Crosslinked Carboxymethyl Rice Starch (CLCMRS)

Crosslinked carboxymethyl rice starch appeared as a white, odorless, poorly flowing powder consistent with the description reported in the previous study [18]. The analysis results pertinent to the characteristics of CLCMRS are compiled in Table 2. 

**Figure 1:**
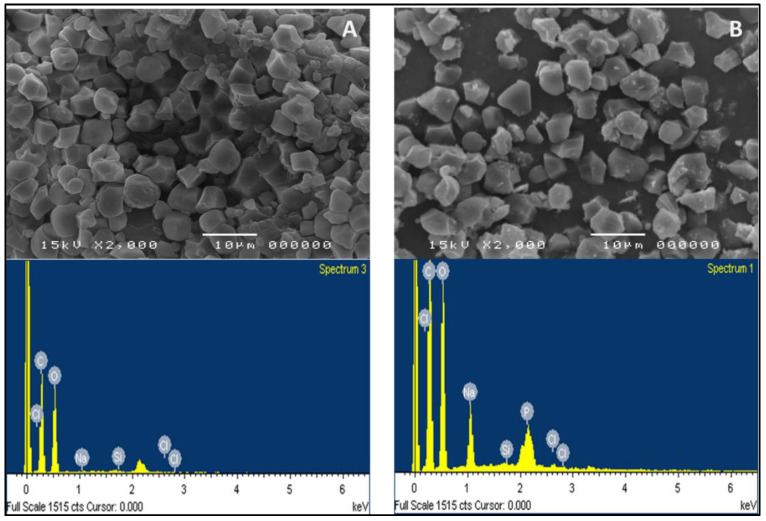
SEM (**top**) and EDX (**bottom**) images of (**A**) native rice starch (NRS) and (**B**) crosslinked carboxymethyl rice starch (CLCMRS) powders. SEM images taken at 2000× magnification.

The degree of substitution of carboxymethyl group (DS), the degree of phosphate crosslinking, water solubility, and swelling power were in line with the characteristics of CLCMRS that were significantly different from those of native rice starch (NRS) [10]. The high moisture content in CLCMRS indicated the hygroscopicity, whereas the high ash content was due to the presence of Na and P from the modification. SEM results (Figure 1) showed that CLCMRS granules were mainly polygonal with diameter sizes ranging from 3 to 6 μm. The granules appeared mostly intact and visually identical in both the shape and the size to those of NRS. The energy-dispersive X-ray (EDX) spectra of NRS and CLCMRS showed the presence of carbon (C) and oxygen (O) as main elemental compositions at the weight ratio of 1.2 to 1 and 1.1 to 1, respectively. In CLCMRS, the increase in the P peak intensity indicated the presence of crosslinked phosphodiester bonds, whereas the increase in Na peak intensity corresponded to the salt formation at the negatively charged oxygen atoms of both the carboxymethyl (-CH_2_C(=O)O) and the phosphodiester (-O-P(=O)(OH)O^−^ groups on the starch chains [17]. 

**Figure 2:**
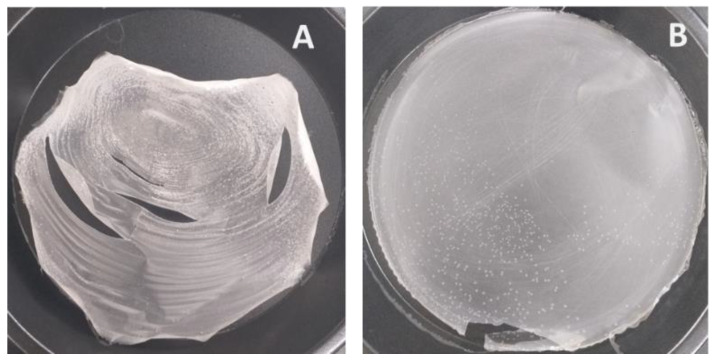
Film-forming property of (**A**) native rice starch and (**B**) crosslinked carboxymethyl rice starch.

Native rice starch, when made fully gelatinized in hot water at 3%*w/v*, can be used to cast a film but the obtained non-plasticized film appeared rigid and brittle (Figure 2A). CLCMRS, on the other hand, was dispersible in unheated water and formed a continuous entangled phase which, upon casting on the Teflon plate and dried in a hot-air oven, yielded an intact, flexible film even without the addition of a plasticizer (Figure 2B). Carboxymethyl starches derived from several starch sources were previously reported to possess a film-forming property [27,28,29], partly because the hydrophilic carboxymethyl groups acting as hydrogen bond disruptors which increased the intermolecular spacing between starch chains, a mechanism mimicking that of a plasticizer [18]. The additional cross-linking reaction, which produced CLCMRS, improved the swelling and disintegrating properties while exerting little or no effect on the film-forming ability. 

### 3.2. Physicochemical Properties of Composite Films 

#### 3.2.1. Average Weight, Film Thickness and Density

Casted by using the solutions of the same concentration (3%*w/v*) and the volume (30 mL), CLCMRS film averaged 10% greater weight compared to HPMC film (Table 3). When combined at various HPMC-CLCMRS ratios without the addition of plasticizer, the weights of the films were in the range of 40.28 ± 0.18 to 40.72 ± 0.33 mg and were not significantly different from one another. All films exhibited uniformed thickness with deviation within the same film strip of less than 15%. CLCMRS film (0.13 ± 0.02 mm) was more than 60% thicker than the HPMC film (0.08 ± 0.01 mm) when casted using the same 3%*w/v* concentration. The thickness of the non-plasticized composite films (0.09 ± 0.01 mm) was not significantly different from that of HPMC, which suggested that the amount of CLCMRS used (up to 20% of the total weight) in the formulations did not significantly alter the thickness of the composite films. As a result, the densities of the non-plasticized films were not significantly different from one another. The addition of glycerol as a plasticizer clearly increased the weight and the thickness of the films. The average weights of the films increased by 2.5–4.0 and 3.5–4.8% when glycerol was added at 1.5 and 2.5%*w/w*, respectively. The film thickness also increased by 11% and 28% with the addition of glycerol at 1.5 and 2.5%*w/w*, respectively. As a result of the higher proportional increase in the film volume than the film weight, the film density decreased as the amount of glycerol was increased. Similar results were observed in the case of cornstarch-based film plasticized with glycerol and sorbitol [22]. Glycerol was reported to affect the thickness of the film by altering the structure of the polymeric chain network such that the free volumes were relocated to form thicker film [30]. The increase was proportional to the amount of glycerol added. 

#### 3.2.2. Moisture Content and Moisture Absorption

The moisture contents of the non-plasticized composite films were slightly higher than that of the HPMC film but were not significantly different among those with varied CLCMRS amounts. A gradual increase in the moisture absorption was observed as the amount of the hygroscopic CLCMRS was increased in the film formulation. The 4:1 HPMC-CLCMRS composite film yielded a 5× higher moisture sorption compared to the HPMC film, although the value remained relatively low (1.16 ± 0.34%). The effect of glycerol concentration on the moisture content, moisture absorption, and film thickness was previously reported [18,30] and the results were consistent with this study. Composite films plasticized by glycerol exhibited a 30–75% increase in moisture content compared to the non-plasticized films, although the increase did not seem to be concentration-dependent. In contrast, glycerol affected the moisture absorption of the films both qualitatively and quantitatively. 

#### 3.2.3. Transparency

The film transparency, evaluated by the calculation of transparency value (T value), indicated HPMC film as the most transparent (7.03 ± 0.77 mm^−1^) among all tested films. The incorporation of the opaque CLCMRS (T value 1.45 ± 0.18 mm^−^^1^) in the formulation caused the composite films to become less transparent. T values decreased gradually as the ratio of CLCMRS in the composite films increased (Table 3). Glycerol, at the concentrations used of 1.5 and 2.5%*w/w*, exhibited no effect on the film transparency. 

### 3.3. SEM of Films

**Figure 3:**
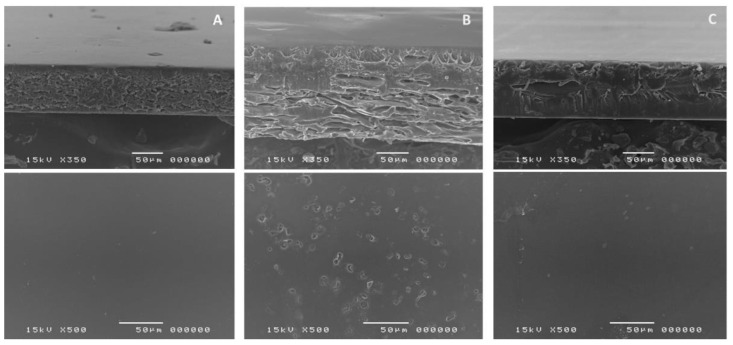
SEM images of the cross-sectional (top) and surface (bottom) of films. (**A**)-HPMC LV5; (**B**)-CLCMRS; (**C**)-Composite HPMC: CLCMRS 83:17 (C-3) Film.

The cross-sectional SEM images showed that HPMC film was formed through a compact orientation of polymer chain with a smooth surface (Figure 3A). This is in agreement with a previous study by Klangmuang & Sothornvit that HPMC-based nanocomposite film formed compact film with minimal free volume within the matrix [31]. In contrast, the texture of CLCMRS film was more loosened and less organized, exhibiting internally expanded space that was likely responsible for the absorption of moisture. Together with a significantly rougher surface due to small pores (Figure 3B), this could explain the higher moisture content recorded for CLCMRS film. The composite film C-3 (Figure 3C) showed the combined characteristic of the two components. The cross-sectional structure remained organized but slightly expanded, and the surface was mostly smooth. Such structure reflected on the higher moisture content and moisture absorption of the composite films. 

### 3.4. FTIR

**Figure 4:**
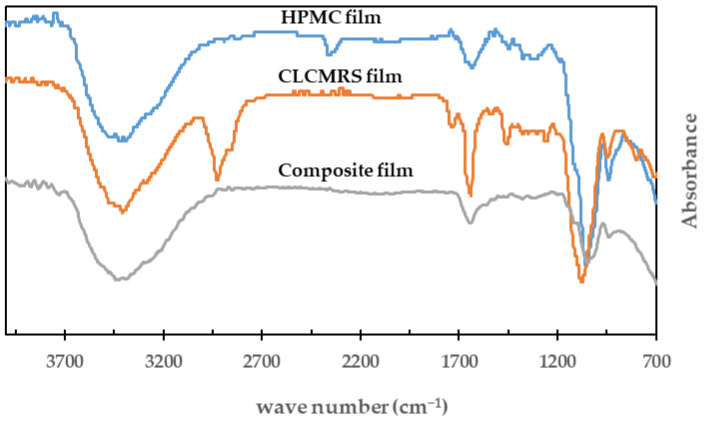
FTIR of HPMC, CLCMRS, and HPMC-CLCMRS composite films.

FTIR spectra of HPMC, CLCMRS, composite films are shown in Figure 4. The band located at the wavenumber 3350–3370 cm^−1^ corresponded to the hydroxyl groups (O-H) of the anhydroglucose unit of both cellulose and starch. The peaks observed at 1640–1650 cm^−1^ were the C-O bond of the six carbon cyclic pyranose. The strong peak at 1650 cm^−1^ of CLCMRS was the characteristic C=O bond of the carboxymethyl group [21]. 

### 3.5. Mechanical Properties

**Figure 5:**
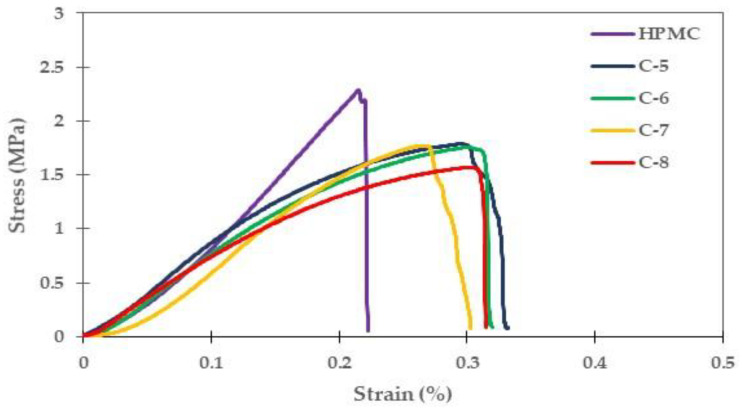
Representative stress-strain curves of the plasticized HPMC-CLCMRS composite films (C-5 to C-8) compared to that of HPMC film.

The tensile strength, elongation at break, and Young’s modulus were determined to evaluate the mechanical properties of the composite films in comparison to pure HPMC. The stress-strain curves of the HPMC film and the glycerol-plasticized composite films with four different HPMC: CLCMRS ratios are shown in Figure 5. The patterns of the pure and composite films are consistent with previous reports [32,33]. The mechanical properties of films are compiled in Table 4. 

#### 3.5.1. Tensile Strength

The tensile test is the most commonly used technique for evaluating the mechanical properties of ODFs [34]. The tensile strength (TS) of HPMC film was recorded at 2.24 ± 0.11 MPa, a 1.5× greater than that of CLCMRS (1.43 ± 0.09 MPa). The results corroborated the film appearances on SEM images, in which the HPMC film was more compact than CLCMRS film. The linear three-dimensional structure of cellulose reinforced the intermolecular hydrogen bonding which allowed it to withstand the applied stress and prevented film break [35]. The intramolecularly bonded helical structures in starch were weaker and the starch film was more likely to rupture. The TS values of the non-plasticized composite films were not significantly different from that of HPMC film, nor were they different from one another, which suggested that the amount of CLCMRS used in this study did not affect the film strength. However, the downward trend of TS as the higher CLCMRS ratio suggested that further increase in CLCMRS composition would eventually cause the TS to decline. This could be explained by the hygroscopicity of CLCMRS which caused an increase in the moisture content of the composite film. A study by Bruni et al. showed a correlation between a high moisture content and a decrease in the tensile strength of the film due to a lower structural stability [23]. A significant increase in the ratio of CLCMRS in the composite could also weaken the cohesion forces of the HPMC film matrix and led to a decrease in TS, which was reported in the case of carboxymethyl cellulose-rice flour composite [21]. The addition of glycerol lowered the TS values of the films, mainly due to a decrease in the interaction of polymer-water molecules [36]. The increase in the moisture content of the film due to the hygroscopic nature of glycerol also contributed to the decrease of TS as the forces between the adjacent polymer chains were reduced. 

#### 3.5.2. Elongation at Break (EAB)

EAB of HPMC film was higher than that of CLCMRS film. This is due to the more extensive hydrogen bonding patterns in cellulose which allowed the exchange and compensated for chain slippage, thus the material was more ductile [36]. The EAB percentages of the non-plasticized composite films were 15–28% lower than that of HPMC film, possibly due to a partial interruption of structural homogeneity of the composite film by the presence of CLCMRS. Glycerol-plasticized films exhibited the expected, concentration-dependent increases in EAB (Table 4). The addition of glycerol into the composite increased the intermolecular repulsions between the polymer chains, creating the free volume in the polymer structure which led to increases in polymer molecular mobility. The improvement in the film flexibility was evidenced by the lowering of Young’s modulus observed in the plasticized films compared to the non-plasticized films. 

### 3.6. Folding Endurance Test

The folding endurance (FE) test has been suggested as a more suitable method for evaluating the actual strength during the production of ODFs [34]. The results showed that HPMC film was more pliable than CLCMRS film. The folding endurance of HPMC film averaged 135 ± 22 times, as opposed to the CLCMRS which broke at an average of 42 ± 12 times (Table 4). The folding endurance of the non-plasticized composite films was dependent on the HPMC: CLCMRS ratio of the composite films. An increase in the CLCMRS amount resulted in a film with less endurance. The thickness of the film also contributed significantly as it was known to negatively affect the folding endurance [36]. A plasticizer improved the flexibility of the films and thus helped increase their folding endurance. Upon an addition of glycerol, the film became flexible and the folding endurance increased significantly to >300 times at a glycerol concentration of 2.5%*w/w.*

### 3.7. In Vitro Disintegration Time

**Figure 6:**
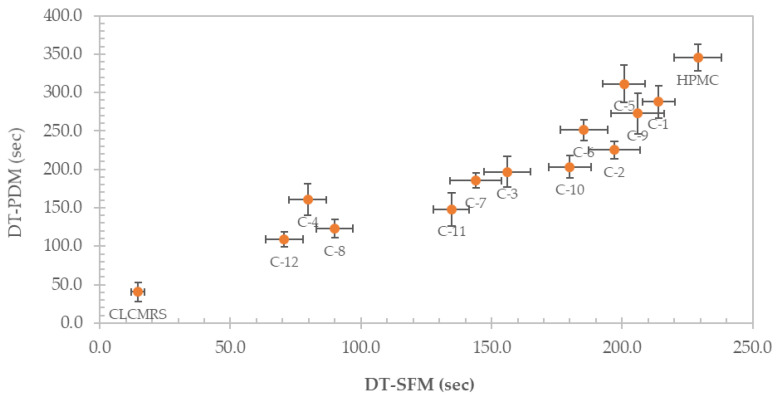
Disintegration times (sec) of HPMC, CLCMRS, and HPMC-CLCMRS composite films determined using the Petri dish method (PDM) and the Slide Frame and Ball method (SFM) and their correlation.

The disintegration times of the films, determined by using two different methods, namely the Petri dish method (PDM) and the slide frame and bead method (SFM), and their correlation are presented in Figure 6. Using PDM, the DT was lowered from 345.3 ± 17.5 s in HPMC film, to between 311.3 ± 24.4 and 272.7 ± 26.4 s in the 9:1 composite, and to as low as 109.0 ± 10.1 s in the 4:1 composite. Similarly, the DT determined by using SFM decreased from 229.0 ± 9.2 sec in HPMC film, to between 214.0 ± 6.2 and 200.7 ± 8.0 s in the 9:1 composite, to as low as 70.7 ±7.1 s in the 4:1 composite. The disintegration time (DT) of the composite films was clearly affected by the presence and the amount of CLCMRS. CLCMRS expedited the film disintegration via water absorption, swelling, and bursting-the three-step mechanism which was well-documented for a modified starch superdisintegrant [37]. The film-forming ability of CLCMRS assured its seamless inclusion into the formulation without putting a burden on HPMC, the primary film former. The addition of glycerol as a plasticizer seemed to also lower the DT, although the effect was less pronounced at low concentrations. DT values obtained from both methods showed a good correlation, with the SFM method yielding a shorter DT on the same sample, which was in agreement with a previously reported study [26]. According to the Ph. Eur., the maximum time limit required for the disintegration of orodisperible tablets was 180 s [38]. This value is currently adopted as a criterion for the DT of ODF since a DT specification for ODF has not yet been established. Composite film formulations with HPMC: CLCMRS ratio of 5:1 (C-3, C-7, C-11) and 4:1 (C-4, C-8, C-12) were suitable for further development of ODFs, whereas formulations C-2, C-6, C-10 could also be tested in a drug-containing formulation, as it was reported that a shorter DT was observed in particle-loaded ODFs compared to particle-free ODFs [26]. 

## 4. Conclusions

Cross-linked carboxymethyl rice starch (CLCMRS), a chemically-modified starch with disintegrating property, was successfully incorporated at various ratios into a hydroxypropylmethylcellulose (HPMC) matrix to form a composite with film-forming properties. The evaluation of the physicochemical and mechanical properties, including weight, thickness, density, appearance and texture, strength, flexibility, elongation, stability, and disintegration, suggested that the HPMC-CLCMRS composites of ratio 4:1 and 5:1 were the ones that maintained the strength and integrity of the HPMC film, while acquiring the rapid disintegration properties of the CLCMRS. With the addition of a plasticizer at 1.5 or 2.5%, the composite films showed decreased tensile strength but increased film elongation and folding endurance. The films disintegrated significantly faster than the HPMC film in two in vitro disintegration models and can be further developed as a film base for the manufacturing of ODF which has become one of the most preferable pharmaceutical dosage forms in recent years. 

## Figures and Tables

**Table 1:** Composition of HPMC-CMRS composite films.

Formulation	Polymer Composition (%)			Glycerol (g/100 g Polymers)
	HPMC E5LV (3 g/100 mL)	CLCMRS (3 g/100 mL)	Ratio	
HPMC	100	-	N*/*A	0
CLCMRS	-	100	N*/*A	0
C-1	90	10	9:1	0
C-2	87.5	12.5	7:1	0
C-3	83.5	16.5	5:1	0
C-4	80	20	4:1	0
C-5	90	10	9:1	1.5
C-6	87	13	7:1	1.5
C-7	83	17	5:1	1.5
C-8	80	20	4:1	1.5
C-9	90	10	9:1	2.5
C-10	87	13	7:1	2.5
C-11	83	17	5:1	2.5
C-12	80	20	4:1	2.5

**Table 2:** Analysis results of crosslinked carboxymethyl rice starch (CLCMRS) powder in comparison with native rice starch (NRS) powder.

Analysis	Unit	CLCMRS	NRS
Total starch	%	81.58 ± 3.87	91.03 ± 3.15
Amylose content	%	18.14	21.23
Moisture content	%	10.60 ± 0.33	7.24 ± 0.12
Ash content	%	6.93 ± 0.08	2.42 ± 0.08
Protein	%	0.00	0.00
Fat	%	0.00	0.00
Degree of carboxymethyl substitution (DS)	-	0.24 ± 0.02	N/A
Degree of phosphate crosslinking (DCx)	-	0.018 ± 0.003	N/A
Water solubility	%	58.5	3.1
Swelling power	g/g	28.43 ± 1.59	2.04 ± 0.18

**Table 3:** Physicochemical properties of HPMC, CLCMRS and their composite films *.

Formulation	Physicochemical Property			Swelling Index (g/g DW)	Moisture Content (%)	Moisture Absorption (%)	Transparency (T Value)
	Average Weight (mg)	Film Thickness (mm)	Density (g/cm ^3^)				
HPMC	40.12 ± 0.11 ^a^	0.08 ± 0.01 ^a^	1.254 ± 0.003 ^a^	3.27 ± 0.16 ^cd^	2.49 ± 0.32 ^a^	0.41 ± 0.02 ^a^	7.03 ± 0.77 ^a^
CLCMRS	44.02 ± 1.41 ^d^	0.13 ± 0.02 ^e^	0.772 ± 0.057 ^e^	15.76 ± 1.89 ^a^	8.50 ± 1.06 ^e^	6.32 ± 0.68 ^e^	1.45 ± 0.18 ^d^
C-1	40.28 ± 0.18 ^ab^	0.09 ± 0.01 ^a^	1.165 ± 0.075 ^ab^	3.87 ± 0.48 ^cd^	3.54 ± 0.22 ^b^	0.75 ± 0.06 ^b^	6.18 ± 0.49 ^a^
C-2	40.68 ± 0.23 ^b^	0.09 ± 0.01 ^a^	1.224 ± 0.082 ^ab^	4.62 ± 1.02 ^cd^	3.72 ± 0.34 ^b^	0.82 ± 0.11 ^b^	5.41 ± 0.77 ^b^
C-3	40.54 ± 0.28 ^ab^	0.09 ± 0.02 ^ab^	1.220 ± 0.074 ^ab^	5.87 ± 0.86 ^bc^	4.05 ± 0.82 ^b^	1.14 ± 0.23 ^b^	5.44 ± 1.02 ^ab^
C-4	40.72 ± 0.33 ^ab^	0.09 ± 0.01 ^a^	1.225 ± 0.072 ^ab^	7.09 ± 0.59 ^b^	4.14 ± 0.75 ^b^	1.16 ± 0.34 ^b^	4.73 ± 0.16 ^b^
C-5	41.89 ± 0.17 ^cd^	0.10 ± 0.03 ^abc^	1.086 ± 0.063 ^bc^	3.95 ± 0.65 ^cd^	5.68 ± 0.68 ^cd^	1.87 ± 0.55 ^bc^	6.52 ± 1.83 ^a^
C-6	42.05 ± 0.39 ^cde^	0.10± 0.01 ^bc^	1.051 ± 0.010 ^c^	4.88 ± 0.23 ^c^	6.19 ± 1.12 ^cde^	2.05 ± 0.46 ^c^	5.79 ± 0.67 ^ab^
C-7	41.93 ± 0.25 ^cd^	0.10± 0.02 ^abcd^	0.984± 0.050 ^c^	6.19 ± 0.35 ^bc^	6.87 ± 0.49 ^de^	2.61 ± 0.56 ^c^	5.21 ± 0.85 ^b^
C-8	41.74± 0.23 ^cd^	0.10 ± 0.02 ^abcd^	0.980 ± 0.049 ^c^	6.96 ± 0.28 ^b^	5.93 ± 0.65 ^cd^	2.98 ± 0.04 ^cd^	5.11 ± 1.26 ^b^
C-9	42.19 ± 0.45 ^cde^	0.11 ± 0.02 ^cde^	1.029 ± 0.111 ^c^	4.05 ± 0.23 ^d^	6.72 ± 0.89 ^cde^	5.13 ± 0.59 ^e^	5.11 ± 1.16 ^b^
C-10	42.62 ± 0.28 ^e^	0.11 ± 0.02 ^cd^	0.969 ± 0.006 ^cd^	5.01 ± 0.74 ^c^	6.98 ± 1.02 ^cde^	4.78 ± 0.76 ^de^	4.74 ± 1.08 ^b^
C-11	42.37 ± 0.50 ^cde^	0.11 ± 0.01 ^d^	0.968 ± 0.077 ^cd^	6.36 ± 0.81 ^bc^	6.32 ± 1.31 ^cde^	5.54 ± 0.97 ^de^	4.40 ± 0.59 ^b^
C-12	41.95 ± 0.31 ^cd^	0.11 ± 0.01 ^cd^	0.927 ± 0.041 ^d^	6.78 ± 1.16 ^b^	5.46 ± 0.64 ^c^	5.32 ± 1.21 ^de^	3.12 ± 0.62 ^c^

* Means followed by different superscript in each column are statistically different (*p* < 0.05).

**Table 4:** Mechanical properties of orodispersible films prepared with HPMC-CMRS composites at different ratio *.

Formulation	Mechanical Property			FE (Times)
	TS (MPa)	EAB (%)	YM (MPa)	
HPMC	2.24 ± 0.11 ^a^	6.03 ± 0.38 ^d^	10.67 ± 1.04 ^b^	135 ± 22 ^c^
CLCMRS	1.43 ± 0.09 ^b^	2.53 ± 0.47 ^g^	20.25 ± 1.57 ^a^	42 ± 12 ^a^
C-1	2.09 ± 0.07 ^a^	4.97 ± 0.19 ^e^	10.93 ± 0.69 ^b^	122 ± 21 ^b,c^
C-2	2.05 ± 0.06 ^a^	5.09 ± 0.44 ^e^	10.28 ± 0.52 ^b^	113 ± 15 ^b,c^
C-3	2.13 ± 0.32 ^a^	4.31 ± 0.53 ^e,f^	11.59 ± 0.90 ^b^	101 ± 18 ^b,c^
C-4	2.01 ± 0.18 ^a^	4.51 ± 0.77 ^e,f^	10.47 ± 0.84 ^b^	96 ± 15 ^b^
C-5	1.61 ± 0.08 ^b^	8.59 ± 0.70 ^b,c^	6.53 ± 0.53 ^d^	266 ± 35 ^d^
C-6	1.75 ± 0.14 ^b^	7.65 ± 0.64 ^c^	7.38 ± 1.77 ^c,d^	251 ± 24 ^d^
C-7	1.65 ± 0.16 ^b^	7.21 ± 0.50 ^c^	7.62 ± 0.64 ^c,d^	233 ± 18 ^d^
C-8	1.57 ± 0.24 ^b^	7.64 ± 0.83 ^c^	8.25 ± 0.55 ^c^	241 ± 13 ^d^
C-9	0.79 ± 0.14 ^c^	10.51 ± 0.58 ^a,b^	4.71 ± 0.63 ^e^	>300 ^e^
C-10	0.66 ± 0.17 ^c^	11.07 ± 0.41 ^a^	4.31 ± 0.42 ^e^	>300 ^e^
C-11	0.72 ± 0.08 ^c^	10.05 ± 0.64 ^a,b^	5.24 ± 0.80 ^d,e^	>300 ^e^
C-12	0.82 ± 0.10 ^c^	9.76 ± 0.57 ^b^	4.99 ± 0.86 ^e^	>300 ^e^

TS-tensile strength; EAB-elongation at break; YM-Young’s modulus; FE-Folding endurance; Values are the average ± standard deviation. * Means followed by different superscript in each column are statistically different (*p* < 0.05).

## References

[1] Ouda G.I., Dahmash E.Z., Alyami H., Iyire A. (2020). A novel technique to improve drug loading capacity of fast/extended release orally dissolving films with potential for paediatric and geriatric drug delivery. AAPS PharmSciTech.

[2] Heinemann R.J.B., Vanin F.M., De Carvalho R.A., Trindade M.A., Fávaro-Trindade C.S. (2017). Characterization of low cost orally disintegrating film (ODF). Polímeros.

[3] Janigová N., Elbl J., Pavloková S., Gajdziok J. (2022). Effects of Various Drying Times on the Properties of 3D Printed Orodispersible Films. Pharmaceutics.

[4] Zakar R.S., Özakar E. (2021). Current overview of oral thin films. Turk. J. Pharm. Sci..

[5] Khalid G.M., Musazzi U.M., Selmin F., Franze S., Minghetti P., Cilurzo F. (2021). Extemporaneous printing of diclofenac orodis-persible films for pediatrics. Drug Dev. Ind. Pharm..

[6] Norfarhana A., Ilyas R., Ngadi N. (2022). A review of nanocellulose adsorptive membrane as multifunctional wastewater treatment. Carbohydr. Polym..

[7] Baghaei B., Skrifvars M. (2020). All-Cellulose Composites: A Review of Recent Studies on Structure, Properties and Applications. Molecules.

[8] Pandey A. (2021). Pharmaceutical and biomedical applications of cellulose nanofibers: A review. Environ. Chem. Lett..

[9] Ghadermazi R., Hamdipour S., Sadeghi K., Ghadermazi R., Asl A.K. (2019). Effect of various additives on the properties of the films and coatings derived from hydroxypropyl methylcellulose—A review. Food Sci. Nutr..

[10] Zhang J., Yang W., Vo A.Q., Feng X., Ye X., Kim D.W., Repka M.A. (2017). Hydroxypropyl methylcellulose-based controlled release dosage by melt extrusion and 3D printing: Structure and drug release correlation. Carbohydr. Polym..

[11] Narváez-Gómez G., Figueroa-Flórez J., Salcedo-Mendoza J., Pérez-Cervera C., Andrade-Pizarro R. (2021). Development and characterization of dual-modified yam (*Dioscorea rotundata*) starch-based films. Heliyon.

[12] Wang L., Liu X., Wang J. (2016). Structural properties of chemically modified Chinese yam starches and their films. Int. J. Food Prop..

[13] Majzoobi M., Pesaran Y., Mesbahi G., Golmakani M.T., Farahnaky A. (2015). Physical properties of biodegradable films from heat-moisture-treated rice flour and rice starch. Starch/Starke.

[14] Bodini R.B., Guimarães J.D.G.L., Monaco-Lourenço C.A., de Carvalho R.A. (2019). Effect of starch and hydroxypropyl methylcellulose polymers on the properties of orally disintegrating films. J. Drug Deliv. Sci. Technol..

[15] Limpongsa E., Jaipakdee N. (2020). Physical modification of Thai rice starch and its application as orodispersible film former. Carbohydr. Polym..

[16] Kittipongpatana O.S., Chaitep W., Kittipongpatana N. (2010). Physicochemical and pharmaceutical properties of cross-linked car-boxymethyl rice starch prepared by a simultaneous dual reaction. Cereal Chem..

[17] Kittipongpatana O.S., Kittipongpatana N. (2022). Physicochemical and Functional Properties of Modified KJ CMU-107 Rice Starches as Pharmaceutical Excipients. Polymers.

[18] Basiak E., Lenart A., Debeaufort F. (2018). How Glycerol and Water Contents Affect the Structural and Functional Properties of Starch-Based Edible Films. Polymers.

[19] Fazeli M., Keley M., Biazar E. (2018). Preparation and characterization of starch-based composite films reinforced by cellulose nanofibers. Int. J. Biol. Macromol..

[20] Teixeira E.D.M., Lotti C., Corrêa A.C., Teodoro K.B.R., Marconcini J.M., Mattoso L.H.C. (2010). Thermoplastic corn starch reinforced with cotton cellulose nanofibers. J. Appl. Polym. Sci..

[21] Kaewprachu P., Jaisan C., Klunklin W., Phongthai S., Rawdkuen S., Tongdeesoontorn W. (2022). Mechanical and Physicochemical Properties of Composite Biopolymer Films Based on Carboxymethyl Cellulose from Young Palmyra Palm Fruit Husk and Rice Flour. Polymers.

[22] Harussani M.M., Sapuan S.M., Firdaus A.H.M., El-Badry Y.A., Hussein E.E., El-Bahy Z.M. (2021). Determination of the Tensile Properties and Biodegradability of Cornstarch-Based Biopolymers Plasticized with Sorbitol and Glycerol. Polymers.

[23] Bruni G.P., de Oliveira J.P., Fonseca L.M., da Silva F.T., Dias A.R.G., da Rosa Zavareze E. (2019). Biocomposite Films Based on Phosphorylated Wheat Starch and Cellulose Nanocrystals from Rice, Oat, and Eucalyptus Husks. Starch-Stärke.

[24] ASTM D 2176-97a (2002). Standard Test Method for Folding Endurance of Paper by the M.I.T. Tester. Annual Book of ASTM.

[25] Kim S., Cho D.-H., Kweon D.-K., Jang E.-H., Hong J.-Y., Lim S.-T. (2020). Improvement of mechanical properties of orodispersible hyaluronic acid film by carboxymethyl cellulose addition. Food Sci. Biotechnol..

[26] Speer I., Steiner D., Thabet Y., Breitkreutz J., Kwade A. (2018). Comparative study on disintegration methods for oral film prepara-tions. Eur. J. Pharm. Biopharm..

[27] Wilpiszewska K. (2019). Hydrophilic films based on starch and carboxymethyl starch. Pol. J. Chem. Technol..

[28] Kittipongpatana O.S., Chaichanasak N., Kanchongkittipoan S., Panturat A., Taekanmark T., Kittipongpatana N. (2006). An Aqueous Film-coating Formulation based on Sodium Carboxymethyl Mungbean. Starch-Stärke.

[29] Rachtanapun P., Thanakkasaranee S., Auras R.A., Chaiwong N., Jantanasakulwong K., Jantrawut P., Phimolsiripol Y., Seesuriyachan P., Leksawasdi N., Chaiyaso T. (2022). Morphology, Mechanical, and Water Barrier Properties of Carboxymethyl Rice Starch Films: Sodium Hydroxide Effect. Molecules.

[30] Tarique J., Sapuan S.M., Khalina A. (2021). Effect of glycerol plasticizer loading on the physical, mechanical, thermal, and barrier properties of arrowroot (Maranta arundinacea) starch biopolymers. Sci. Rep..

[31] Klangmuang P., Sothornvit R. (2016). Barrier properties, mechanical properties and antimicrobial activity of hydroxypropyl methylcellulose-based nanocomposite films incorporated with Thai essential oils. Food Hydrocoll..

[32] Shi S.-C., Chen T.-H., Mandal P.K. (2020). Enhancing the Mechanical and Tribological Properties of Cellulose Nanocomposites with Aluminum Nanoadditives. Polymers.

[33] Prado N.R.T., Raabe J., Mirmehdi S., Hugen L.N., Lima L.C., Ramos A.L.S., Junior M.G., Tonoli G.H.D. (2017). Strength im-provement of hydroxypropyl methycellulose starch films using cellulose nanoncrystal. Cerne.

[34] Takeuchi Y., Ikeda N., Tahara K., Takeuchi H. (2020). Mechanical characteristics of orally disintegrating films: Comparison of folding endurance and tensile properties. Int. J. Pharm..

[35] Sun S., Mitchell J.R., MacNaughtan W., Foster T., Harabagiu V., Song Y., Zheng Q. (2009). Comparison of the Mechanical Properties of Cellulose and Starch Films. Biomacromolecules.

[36] Mahadevaiah, Shivakumara L.R., Demappa T., Singh V. (2017). Mechanical and barrier properties of hydroxypropylmethylcellulose edible polymer films with plasticizer combinations. J. Food Process. Preserv..

[37] Markl D., Zeitler J.A. (2017). A Review of Disintegration Mechanisms and Measurement Techniques. Pharm. Res..

[38] Conseil de L’Europe (2020). European Pharmacopoeia, 10th Ed. Eur. Pharm..

